# Elevator Car Vibration Signal Denoising Method Based on CEEMD and Bilateral Filtering

**DOI:** 10.3390/s22176602

**Published:** 2022-09-01

**Authors:** Dapeng Niu, Jiaqi Wang

**Affiliations:** College of Information Science and Engineering, Northeastern University, Shenyang 110819, China

**Keywords:** vibration signal, denoising, CEEMD, bilateral filtering

## Abstract

Elevator car vibration signals are important information to monitor and diagnose the operating status of elevators, but during the process of vibration signals acquisition, vibration signals are always inevitably disturbed by noise, which affects further research. Therefore, aiming at the vibration signal with noise, we propose a new vibration signal denoising method on the basis of complementary ensemble empirical mode decomposition (CEEMD) and bilateral filtering. Firstly, the collected original vibration signals are decomposed by the CEEMD into several inherent mode functions. Then, the false components are removed by determining the correlation coefficients of mode components, and then the noise-dominate components are denoised by bilateral filtering. Finally, the processed inherent mode functions are reconstructed to require the denoised signal. We test the method through simulation and practical application. The results indicate that the proposed method can efficaciously reduce the noise in the vibration signal of an elevator car.

## 1. Introduction

With the development of the city, various high-rise buildings continue to appear. As a kind of transportation equipment, the elevator plays a vital role in these modern buildings, but it is inevitable that some faults occur in the process of putting the elevator into operation. If we can take measures to diagnose the failure of the elevator in a shorter time, we can reduce the losses. Vibration signals are often used to judge the fault type, but the vibration signals we generally collect are mixed with various noises, so we need to eliminate noise on the premise of ensuring that the pure signals are not affected.

Fourier transform is a frequently used method to denoise vibration signals. Under the influence of the Fourier transform, the vibration signal can shift from the time-domain state with time as the abscissa to the frequency-domain state with frequency as the abscissa. The process is actually the decomposition process of the signal, and the original signal finally becomes the combination of multiple sine waves. The premise of the Fourier transform application is that the signal processed is the stationary signal, but the signal we usually obtain is not a stationary signal, so it is difficult to obtain satisfactory results by using Fourier transform. Different from Fourier transform, wavelet transform can place the signal on the coordinate axis with time as the abscissa and frequency as the ordinate. Based on the wavelet transform theory of vibration signal, many denoising methods are proposed. The modulus maximum denoising method [1] calculates the modulus maximum of the signal after wavelet transformation, selects an appropriate threshold, retains the wavelet coefficient of the corresponding extreme points, and carries out signal reconstruction to achieve denoising. The spatial correlation denoising method [2] takes the scale correlation of signals as the key factor of denoising and determines whether to retain the coefficients according to the similarity between scales. The wavelet threshold denoising [3] sets the judgment threshold based on experience or other methods. By comparison and analysis, the coefficients smaller than the threshold are removed, and the coefficients larger than the threshold are retained. Finally, the purpose of denoising is achieved through wavelet inverse transform.

With the development of wavelet denoising, wavelet packet denoising methods have emerged, and there are more and more improved methods based on wavelet packet denoising. Li et al. [4] realized the signal denoising by setting the threshold to a variable value on the basis of wavelet packet decomposition of the signal. Chen et al. [5] combined sparse knowledge with wavelet packet decomposition to remove noise in the vibration signal, and the sparse knowledge used can restore wavelet packet coefficients of the pure signal to a large extent. Regardless of wavelet denoising or wavelet packet denoising, it is necessary to choose the decomposition layers and wavelet basis, but the selection of these two parameters usually needs to be based on experience once improper selection will affect the final denoising effect of the vibration signal.

In addition to wavelet analysis, Huang et al. [6] presented empirical mode decomposition (EMD) for the first time, which can represent a signal as the sum of multiple modal components and a residual component. Later, this method was applied in vibration signal denoising [7]. However, from the perspective of practical application effect, if signal is decomposed by using empirical mode decomposition, there are false components and aliasing components in the obtained components. Based on the problems existing in empirical mode decomposition, Wu et al. [8] put forward ensemble empirical mode decomposition (EEMD). The method greatly reduces the aliasing components in the decomposition process, but the operation of adding white noise makes the residual noise still exist in the reconstructed signal. Later, Yeh et al. [9] suggested complementary ensemble empirical mode decomposition (CEEMD), which greatly reduces the aliasing components. After that, a series of the improved method has been proposed to overcome the shortcomings of EMD and its improved algorithms. Dang et al. [10] introduced the method of using EMD and wavelet transform simultaneously in vibration signal denoising. Chegini et al. [11] combined EMD and wavelet analysis and applied them to denoise in bearing vibration signals. Because wavelet packet transform can achieve better analysis of medium- and high-frequency components of signals, the vibration signal denoising method combining CEEMD and wavelet packet according to the different characteristics of noise and pure signals in vibration signals was proposed in [12,13,14,15,16,17]. The above improved methods mainly combined empirical mode decomposition or its improved algorithms with wavelet decomposition or wavelet packet decomposition for denoising. The denoising process would inevitably involve the selection of wavelet base and the determination of decomposition layers. Therefore, there might be the same problems with wavelet denoising and wavelet packet denoising. In 2014, Dragomiretskiy and Zosso [18] proposed the VMD algorithm, which can decompose the signal into AM and FM component signals. Later VMD algorithm was applied to signal denoising. Long et al. [19] proposed a denoising method combining the VMD algorithm and wavelet decomposition. Yu et al. [20] applied the VMD algorithm to seismic signal denoising. The algorithm has better decomposition precision for complex data, but the number of decomposition layers and penalty factors need to be set in advance before using VMD. Once these two parameters are set improperly, the final decomposition effect will be greatly affected.

At present, Image denoising methods commonly used include gaussian filtering, median filtering, average filtering, and so on. These methods are denoising methods for two-dimensional signals, and some of them have a good filtering effect on noise points. Therefore, we can try to transform these methods with better denoising performance to make them suitable for one-dimensional vibration signals. The Gaussian filtering method has a good denoising effect for random Gaussian noise in the image, but it will affect the edge information of the image in the process of denoising so that the final image after denoising is not clear enough. The median filtering method has a good denoising effect on isolated noise and does not affect the edge information of the image. However, the method needs to determine the size of the sliding window according to the actual situation. Once the improper selection is made, the final image after denoising will become blurred. Average filtering affects the restoration of image edges while removing noise. Bilateral filtering is an evolutionary algorithm of gaussian filtering, which not only considers spatial proximity but also involves the similarity of pixel values. The method is a kind of nonlinear filtering technology that can realize edge preserving and denoising. At present, many images are denoised using a bilateral filtering algorithm [21,22,23].

The noise in the elevator car comes from the car itself and the external environment, and its frequency is mainly medium and low frequency. For the noise filtering in the elevator signal, this paper proposes a denoising method combining bilateral filtering and CEEMD. In the new method, CEEMD and bilateral filtering are combined for the first time to denoise the elevator car vibration signal. Firstly, CEEMD is used to decompose the elevator vibration signal, and the decomposition result is multiple modal components and a residual component. The paper mainly carries out further processing and analysis of the decomposed modal components. The correlation coefficient and multi-scale arrangement entropy are used in the processing, and then the components dominated by noise and the components dominated by the signal are determined. Because bilateral filtering has a good filtering effect on medium- and low-frequency signal, it is applied to the components dominated by noise to remove the noise in the elevator vibration signal. Then, the signal is reconstructed to obtain the denoised signal. Finally, the method is applied to the simulation signal and the elevator vibration signal, respectively, which verifies its superiority and shows that it has a better denoising effect.

The overall structure of this paper is as follows: Section 2 introduces empirical mode decomposition and its improved algorithm and variational mode decomposition. Section 3 introduces the method we suggest in this paper in detail. Section 4 applies the method to the simulation analysis of elevator car vibration signals. Section 5 applies the method, EMD denoising, EEMD denoising, CEEMD denoising, bilateral filtering denoising, and VMD denoising to the elevator car vibration signal and compares the final denoising effect of different methods. The last part draws the conclusion of the whole paper.

## 2. Theoretical Background

### 2.1. Empirical Mode Decomposition (EMD)

The formula of empirical mode decomposition is as follows:

(1) $$\begin{array}{c} x\left(t\right)=\overset{n}{\underset{i=1}{\sum }}imf_{i}+r\left(t\right) \end{array}$$

where $imf_{i}$ is ith inherent modal function, and r(t) is the residual component. 

Each modal component needs to meet the following requirements [24]:

(1) The difference between the number of local extremum points and zeros crossing of the component is 0 or 1;

(2) The upper envelope curve and the lower envelope curve in the component are composed of maximum points and minimum points, respectively, and one-half of their sum equals zero.

The steps of empirical mode decomposition are as follows:

Step 1: Determine all maximum and minimum points of the original signal x(t).

Step 2: According to the maximum and minimum points found in Step1, selecting the cubic spline function to fit them respectively requires the upper envelope U(t) and the lower envelope D(t), and the mean value m(t) of the two envelopes can be obtained by formula (2).

(2) $$m\left(t\right)=\frac{U\left(t\right)+D\left(t\right)}{2}$$

Step 3: Remove the mean envelope m(t) from the input signal x(t). The final obtained value is denoted as $h_{1}\left(t\right)$, $h_{1}\left(t\right)=x\left(t\right)-m\left(t\right)$. If $h_{1}\left(t\right)$ satisfies the condition that the modal component must have, $h_{1}\left(t\right)$ is an inherent modal function, denoted as $c_{1}\left(t\right)$, and proceed with step4. Otherwise, let $x\left(t\right)=h_{1}\left(t\right)$, and proceed with step1–step3.

Step 4: Calculation of residual signal $r_{1}\left(t\right)$:

(3) $$\begin{array}{c} r_{1}\left(t\right)=x\left(t\right)-c_{1}\left(t\right) \end{array}$$

Step 5: Let $x\left(t\right)=r_{1}\left(t\right)$, and begin execution from step1 until the next modal component $c_{2}\left(t\right)$ is found. After n times of repeated iterations, all modal components and one residual component $r_{n}\left(t\right)$ are finally obtained:

(4) $$\{\begin{array}{c} r_{1}\left(t\right)-c_{2}\left(t\right)=r_{2}\left(t\right)\\ r_{2}\left(t\right)-c_{3}\left(t\right)=r_{3}\left(t\right)\\ .\\ .\\ .\\ r_{n-1}\left(t\right)-c_{n}\left(t\right)=r_{n}\left(t\right) \end{array}$$

### 2.2. Ensemble Empirical Mode Decomposition (EEMD)

Ensemble empirical mode decomposition is a modified algorithm for the shortcomings of EMD. If the signal varies greatly in different time scales, the modal components obtained by empirical mode decomposition may contain more than the information of the current time scale, which is called mode aliasing. In order to reduce this phenomenon, EEMD adds white noise on the basis of unprocessed signals. Because the added signal is characterized by uniform spectrum distribution, all the signals obtained after ensemble empirical mode decomposition will be placed on the corresponding time scale. The steps of Ensemble empirical mode decomposition are shown below [8]:

Step 1: Add white noise to the original signal;

Step 2: The signal after processing in Step 1 is disassembled into a combination of IMF components by empirical mode decomposition (EMD);

Step 3: Repeat Step1 and Step2 n times;

Step 4: Take the IMF aggregate mean obtained each time as the final result.

### 2.3. Complementary Ensemble Empirical Mode Decomposition (CEEMD)

CEEMD improves the modal aliasing problem in the signal processing process. The difference between this method and EEMD is that the latter adds multiple white noises to the signal before decomposition, while the former adds pairs of white noise with opposite signs to the signal before decomposition, which can not only decrease the effect of noise on the decomposition result but also enhance the computational efficiency.

The main steps of CEEMD are as follows [25]:

Step 1: Add a pair of noise signals to the input signal, and the added white noise has the same amplitude.

(5) $$\left[\begin{array}{c} M_{1}^{i}\\ M_{2}^{i} \end{array}\right]=\left[\begin{array}{cc} 1 & 1\\ 1 & -1 \end{array}\right]\left[\begin{array}{c} X\\ N_{i} \end{array}\right]$$

where X is the original input signal, and $N_{i}$ is the white noise added for the ith time.

Step 2: After adding white noise for the ith time, EMD decomposition of $M_{1}^{i}$ and $M_{2}^{i}$ is performed to require the corresponding components $c_{1,j}^{i}$ and $c_{2,j}^{i}$, as well as the corresponding residuals $r_{1}^{i}$ and $r_{2}^{i}$.

Step 3: Take the average values of modal components and residuals, respectively.

(6) $$c_{j}^{i}=\frac{1}{2}\left(c_{1,j}^{i}+c_{2,j}^{i}\right)$$

(7) $$r^{i}=\frac{1}{2}\left(r_{1}^{i}+r_{2}^{i}\right)$$

Step 4: Similarly, if n pairs of white noise with the opposite sign are added, the final IMF is:

(8) $$c_{j}=\frac{1}{n}\overset{n}{\underset{i=1}{\sum }}c_{j}^{i}$$

### 2.4. Variational Mode Decomposition (VMD)

The core of variational mode decomposition is to construct and solve variational problems, which is an adaptive, completely non-recursive signal processing method. The method determines the frequency center and bandwidth of each component by iteratively searching for the optimal solution of the variational model in the process of obtaining the decomposed components so that the frequency domain division of the signal and the effective separation of each component can be adaptively realized.

The variational mode decomposition can decompose the signal into multiple amplitude-frequency modulated signals, and the decomposition formula is as follows:

(9) $$f\left(t\right)=\overset{K}{\underset{k=1}{\sum }}u_{k}\left(t\right)$$

(10) $$u_{k}\left(t\right)=A_{k}\left(t\right)\cos\left(\varphi_{k}\left(t\right)\right)$$

where f(t) is the original signal and $u_{k}\left(t\right)$ is the kth harmonic signal, $A_{k}\left(t\right)$ is the amplitude of the kth harmonic signal, $\varphi_{k}\left(t\right)$ is the phase of the kth component, and *K* is the number of modal components obtained by decomposition.

The specific decomposition steps of the variational modal decomposition algorithm are as follows [26,27]:

(1) Hilbert transform for each modal signal is as follows:

(11) $$\left(\delta \left(t\right)+\frac{j}{\pi t}\right)*u_{k}\left(t\right)$$

where δ(t) is the dirac distribution, *j* is the imaginary part, ∗ is the convolution symbol. 

(2) The spectrum of each mode is modulated to the corresponding fundamental frequency band.

(12) $$\left[\left(\delta \left(t\right)+\frac{j}{\pi t}\right)*u_{k}\left(t\right)\right]e^{-jw_{k}t}$$

where $w_{k}$ represents the center frequency of the kth mode component.

(3) Calculate the square root of the L2 norm gradient of the demodulated signal and estimate the bandwidth of each modal signal. The variational problem is expressed as follows:

(13) $$\begin{array}{c} min\\ \left\{u_{k}\right\},\left\{w_{k}\right\} \end{array}\left\{\underset{k}{\sum }\|\partial_{t}\left[\left(\delta \left(t\right)+\frac{j}{\pi t}\right)*u_{k}\left(t\right)\right]e^{-jw_{k}t}{\|}_{2}^{2}\right\}\;s.t.\underset{k}{\sum }u_{k}\left(t\right)=f$$

where $\partial_{t}$ represents the partial derivative of the function with respect to time, δ(t) is the unit pulse function.

The quadratic penalty factor α and the lagrangian penalty operator λ(*t*) are introduced to convert the constrained problem into the unconstrained problem. The extended Lagrangian expression is as follows:

(14) $$\begin{array}{c} L\;\left(\left\{u_{k}\right\},\left\{w_{k}\right\},\lambda \right)=\alpha \underset{k}{\sum }\|\partial_{t}\left[\left(\delta \left(t\right)+\frac{j}{\pi t}\right)*u_{k}\left(t\right)\right]e^{-jw_{k}t}{\|}_{2}^{2}\\ +\|f\left(t\right)-\underset{k}{\sum }u_{k}\left(t\right){\|}_{2}^{2}+\langle \lambda \left(t\right),f\left(t\right)-\underset{k}{\sum }u_{k}\left(t\right)\rangle \end{array}$$

where α represents the bandwidth parameter.

The Alternating Direction Method of Multipliers (ADMM) is used to solve the variational problem. By updating $u_{k}^{n+1}$, $w_{k}^{n+1}$ and $\lambda^{n+1}$, we can find the Lagrangian saddle point, obtain the optimal solution of $u_{k}^{n+1}\left(w\right)$ and $w_{k}^{n+1}\left(w\right)$, and substitute them into ADMM to obtain a complete VMD algorithm. The specific steps are as follows:

Step 1: Initialize $u_{k}^{1}$, $w_{k}^{1}$, $\lambda^{1}$ and initializes n to 0.

Step 2: Update $u_{k}$, $w_{k}$.

(15) $$\begin{array}{l} u_{k}^{n+1}\left(w\right)\\ =\frac{f\left(w\right)-\sum_{i=1,i<k}^{K}u_{i}^{n+1}\left(w\right)-\sum_{i=1,i<k}^{K}u_{i}^{n}\left(w\right)+\frac{\lambda^{n}\left(w\right)}{2}}{1+2\alpha {\left(w-w_{k}^{n}\right)}^{2}} \end{array}$$

(16) $$w_{k}^{n+1}=\frac{\int_{0}^{\infty }w{\left|u_{k}^{n+1}\left(w\right)\right|}^{2}dw}{\int_{0}^{\infty }{\left|u_{k}^{n+1}\left(w\right)\right|}^{2}dw}$$

Step 3: Update λ.

(17) $$\lambda^{n+1}\left(w\right)=\lambda^{n}\left(w\right)+\tau \left[f\left(w\right)-\overset{K}{\underset{k=1}{\sum }}u_{k}^{n+1}\left(w\right)\right]$$

where τ represents the noise tolerance parameter. When the signal contains strong noise, we can set *τ* = 0.

Step 4: Repeat Step 2 and Step 3 until the following iteration constraints are satisfied.

(18) $$\overset{K}{\underset{k=1}{\sum }}\frac{\|u_{k}^{n+1}-u_{k}^{n}{\|}_{2}^{2}}{\|u_{k}^{n}{\|}_{2}^{2}}<\varepsilon$$

where $\varepsilon =10^{-6}$.

## 3. Vibration Signal Denoising Method Based on CEEMD 

**Figure 1:**
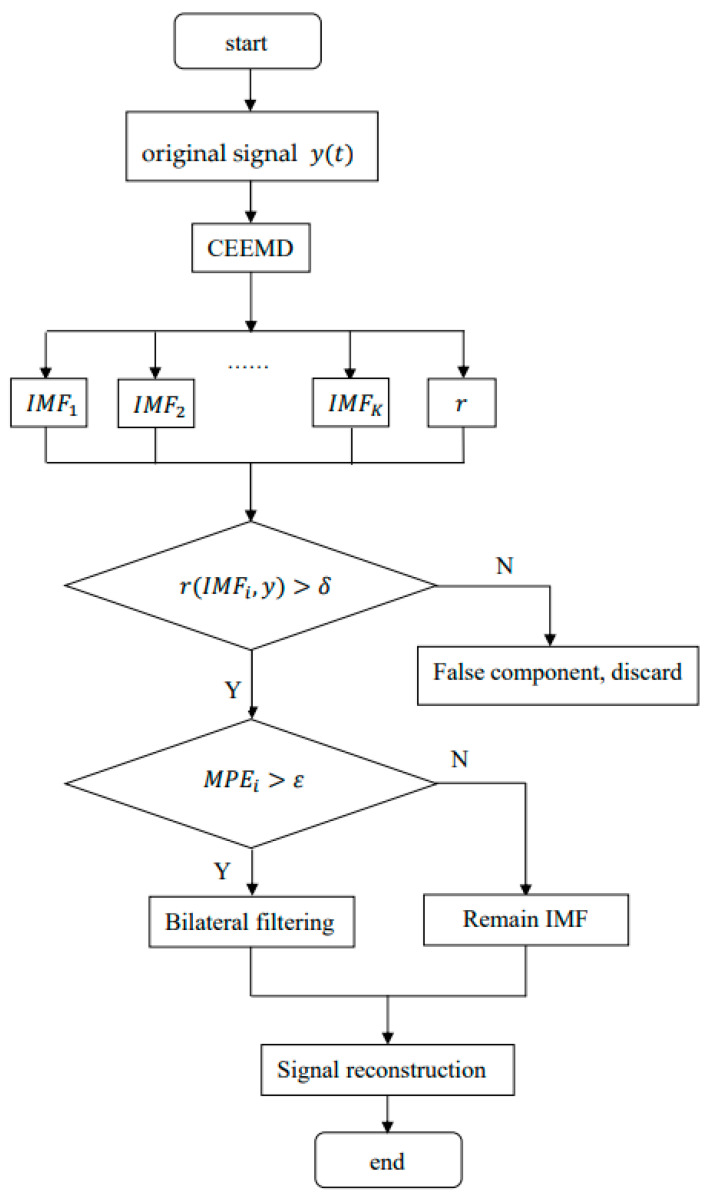
Vibration signal denoising flow chart combining CEEMD and bilateral filtering.

In the paper, we take advantage of the CEEMD to decompose the vibration signal into several intrinsic mode functions. Then remove the false components by calculating the correlation coefficient of the IMFs. Because there is still noise in the remaining signal, the remaining signal is divided into two parts by multi-scale permutation entropy, and then the signal in the dominant position of noise is denoised by bilateral filtering. Finally, the processed signal is refactored to require the final denoising Signal. Figure 1 shows the vibration signal denoising flow chart combining CEEMD and bilateral filtering.

The main steps of the new method are as follows:

(1) Let the noisy signal be y(t), and divide the signal into several intrinsic modal functions and one residual component through CEEMD.

(19) $$y\left(t\right)=\overset{n}{\underset{i=1}{\sum }}imf_{i}\left(t\right)+r_{n}\left(t\right)$$

(2) Determine the correlation coefficient of each component and the original signal and distinguish between false component and true component. The correlation coefficient quantitatively describes the degree of correlation between each modal component and the original signal. The larger the correlation coefficient of the modal component is, the more similar it is to the original signal. The smaller the correlation coefficient of the modal component, the greater the difference between the modal component and the original signal. if the correlation coefficient of the modal component is small, it indicates that the modal component is a false component, which needs to be discarded, and the remaining IMFs are reserved. In this paper, our research object is the elevator car vibration signal, and the experiments involved are also only related to the characteristics of the elevator vibration signal. Therefore, considering the results of multiple denoising experiments for the elevator signal, we set the threshold of the correlation coefficient to 0.1~0.2 of the maximum correlation coefficient. Because the paper does not carry out experimental verification on other equipment except the elevator, the current setting of this threshold is only applicable to the vibration signal of the elevator car. δ in Figure 1 indicates the threshold of the correlation coefficient.

(20) $$\rho_{i}=\frac{\int_{-\infty }^{+\infty }imf_{i}\left(t\right)y\left(t\right)dt}{\sqrt{\int_{-\infty }^{+\infty }imf_{i}{\left(t\right)}^{2}dt\sqrt{\int_{-\infty }^{+\infty }y{\left(t\right)}^{2}dt}}}$$

where $\rho_{i}$ is the correlation coefficient between the $imf_{i}$ and the input signal, $\left|\rho_{i}\right|\leq 1$, i=1……n.

(3) Determine the multi-scale permutation entropy of the remaining IMF components and divide the signals into the signal-dominated IMF components and the noise-dominated IMF components. Multi-scale permutation entropy is the permutation entropy of a signal at different scales. Multi-scale permutation entropy can represent the characteristics of signals at different scales, determine the complexity and randomness of signals, and highlight the small changes in signals. It is an important parameter for analyzing and processing vibration signals generated by mechanical equipment. The process is to coarse-grain the original time series to construct a multi-scale time series and then calculate the permutation entropy at each scale. The smaller the permutation entropy is, the more regular the time series is. The higher the permutation entropy, the more complex the time series. Therefore, we calculate the multi-scale permutation entropy of IMF components to determine their categories.

The calculation steps of multi-scale arrangement entropy of each modal component are as follows:

Let the time series of each modal component be X={x(i),i=1,2,3,……,n}, where n is the number of sampling points.

Step 1: The time series are coarsely granulated to obtain the processed sequences.

(21) $$y^{s}\left(j\right)=\frac{1}{s}\overset{js}{\underset{i=\left(j+1\right)s+1}{\sum }}x\left(i\right)$$

where s is the scale factor, and $y^{s}\left(j\right)$ is the time series under different scale factors.

Step 2: Reconstruct the time series $y^{s}\left(j\right)$.

(22) $$Y_{t}^{s}=\left\{y_{t}^{s},y_{t+\tau }^{s},\ldots ,y_{t+\left(m-1\right)\tau }^{s}\right\}$$

where m stands for embedding dimension, and τ stands for delay time. If the above formula is sorted in ascending order, there are m! permutations in multi-scale order. The probabilities of time series at each scale are calculated:

(23) $$P_{t}^{s}=\frac{K}{\frac{n}{s}-m+1}$$

where K represents the number of occurrences of each type, and the permutation entropy of each scale is:

(24) $$H_{P}^{s}=-\overset{m!}{\underset{i=1}{\sum }}P_{t}^{s}lnP_{t}^{s}$$

Normalize the above equation:

(25) $$h_{P}^{s}=\frac{H_{P}^{s}}{\ln\left(m!\right)}$$

The calculated multi-scale permutation entropy of the IMF component is denoted as MPE(i),i=1,2,…,q. q represents the number of IMF components.

If MPE(i) is large, it indicates that the IMF component corresponding to this value is a noise-dominated component. If MPE(i) is small, the IMF component corresponding to this value is the signal-dominated component. The experimental object of this paper is the elevator car vibration signal. After many tests, it is more appropriate to set the threshold value of MPE between 0.7 and 0.8. ε in Figure 1 indicates the threshold of the MPE. Similar to δ, the value of ε we selected this time is only applicable to the elevator car vibration signal. 

(4) Keep the signal-dominated components and remove noise from components containing a lot of noise by bilateral filter.

Bilateral filtering is a nonlinear filter, which is usually used to process image noise. When processing adjacent pixel values, it takes into account the proximity relationship in the distance and the similarity in gray scale at the same time. It realizes adaptive filtering by nonlinear combination of spatial proximity and pixel value similarity [28]. The expression of bilateral filter is as follows [29]:

(26) $$g\left(u\right)=\frac{\sum_{\left(x,y\right)\epsilon M_{i,j}}w\left(i,j,x,y\right)f\left(x,y\right)}{\sum_{\left(x,y\right)\epsilon M_{i,j}}w\left(i,j,x,y\right)}$$

(27) $$w\left(i,j,x,y\right)=w_{d}\left(i,j\right)*w_{r}\left(i,j\right)$$

(28) $$w_{d}\left(i,j\right)=e^{-\frac{{\left|i-x\right|}^{2}+{\left|i-y\right|}^{2}}{2\sigma_{d}^{2}}}$$

(29) $$w_{r}\left(i,j\right)=e^{-\frac{{\left|f\left(i,j\right)-f\left(x,y\right)\right|}^{2}}{2\sigma_{r}^{2}}}$$

where g(u) is the image after bilateral filtering, $M_{i,j}$ indicates the pixel set of (2N+1)×(2N+1) spatial neighborhood centered on (i,j), f(x,y) indicates the pixel value of (x,y) in $M_{i,j}$, $w_{d}\left(i,j\right)$ is the spatial proximity Gaussian function, $w_{r}\left(i,j\right)$ is the pixel value similarity Gaussian function, $\sigma_{d}$ is the spatial distance standard deviation, and $\sigma_{r}$ is the numerical similarity standard deviation.

The signal studied in the paper is one dimension vibration signal. In order to illustrate how to apply bilateral filtering to vibration signal, the following example is given [30]:

Given a vibration signal *Y* with noise, where S is the noiseless signal and *N* is the noise signal.

(30) Y=S+N

For this vibration signal, the bilateral filtering formula is changed as follows, which represents the normalized weighted average value of neighborhood points with the size of 2R + 1:

(31) $$S\left(\xi \right)=\frac{\sum_{x=-R}^{R}W\left[\xi ,x\right]\gamma \left[\xi -x\right]}{\sum_{x=-R}^{R}W\left[\xi ,x\right]}$$

where γ[ξ−x] represents the amplitude of the vibration signal at ξ−x, W[ξ,x] is the weight coefficient of bilateral filtering, which is the product of $W_{d}\left[\xi ,x\right]$ and $W_{r}\left[\xi ,x\right]$. The formulas for the two parameters are as follows:

(32) $$W_{d}\left[\xi ,x\right]=e^{-\frac{d^{2}\left(\left|\xi \right|,\left|\xi -x\right|\right)}{2\sigma_{d}^{2}}}=e^{-\frac{x^{2}}{2\sigma_{d}^{2}}}$$

(33) $$W_{r}\left[\xi ,x\right]=e^{-\frac{d^{2}\left(\gamma \left[\xi \right],\gamma \left[\xi -x\right]\right)}{2\sigma_{r}^{2}}}=e^{-\frac{\gamma \left[\xi \right]-\gamma {\left[\xi -x\right]}^{2}}{2\sigma_{r}^{2}}}$$

(5) Finally, reconstruct the processed signal to obtain the denoising signal $y^{\prime }\left(t\right)$. According to the above description, Algorithm 1 summarizes the new denoising method proposed in the paper.

**Algorithm 1:** The workflow of new method**Input:**y(t), δ, ε **Output:**$y^{\prime }\left(t\right)$ Procedure: Decompose y(t) to n modes by CEEMD the decomposed modes are denoted as $imf_{i}\left(t\right)\;\left(i=1,2,\ldots \ldots n\right)$ **For**
i=1:n Determine the correlation coefficient between $imf_{i}\left(t\right)$ and the original signal the correlation coefficient is denoted as $\rho_{i}$ **If**
$\rho_{i}<\delta$  Discard $imf_{i}\left(t\right)$ Else  Determine the multi-scale arrangement entropy of $imf_{i}\left(t\right)$  the multi-scale arrangement entropy is denoted as MPE(i)  **If**
MPE(i)>ε()   Bilateral filtering is needed to denoise  Else   Remain original $imf_{i}\left(t\right)$ Reconstruct the denoised signal $y^{\prime }\left(t\right)$.

## 4. Simulation Signal Verification

Because the frequency of elevator vibration signal is usually 0.5~80 Hz, we set an analog signal to simulate its vibration signal and add Gaussian white noise to the analog signal as the noise in the signal. The formula for obtaining the signal is as follows:

(34) Y=X+N

(35) $$X=A*\overset{n}{\underset{i=1}{\sum }}amp\left(i\right)*\sin\left(2*\pi *f\left(i\right)*t\right)$$

where *Y* is the signal with added noise, *X* is the original signal, *N* is the gaussian white noise signal, and A is a constant value. amp(i) is the corresponding amplitude of vibration signal with frequency of f(i), respectively: 13, 3, 4, 8, 5, 7, 4, and 3; f is the frequency of vibration signals, respectively: 10, 20, 30, 40, 50, 60, 70, and 80.

**Figure 2:**
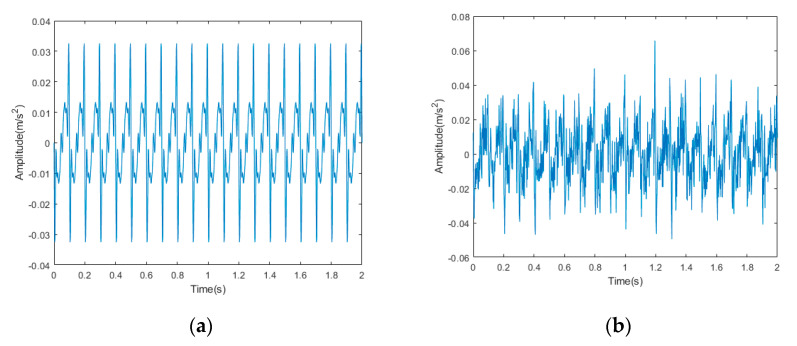
Time domain signal diagram: (**a**) original signal; (**b**) noisy signal.

**Figure 3:**
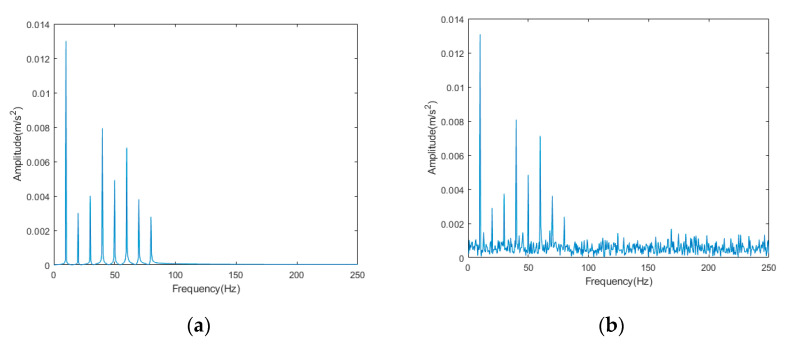
Frequency domain signal diagram: (**a**) original signal; (**b**) noisy signal.

The time domain signal diagram of the original signal and the noisy signal is shown in Figure 2, and the frequency domain signal diagram is shown in Figure 3.

**Figure 4:**
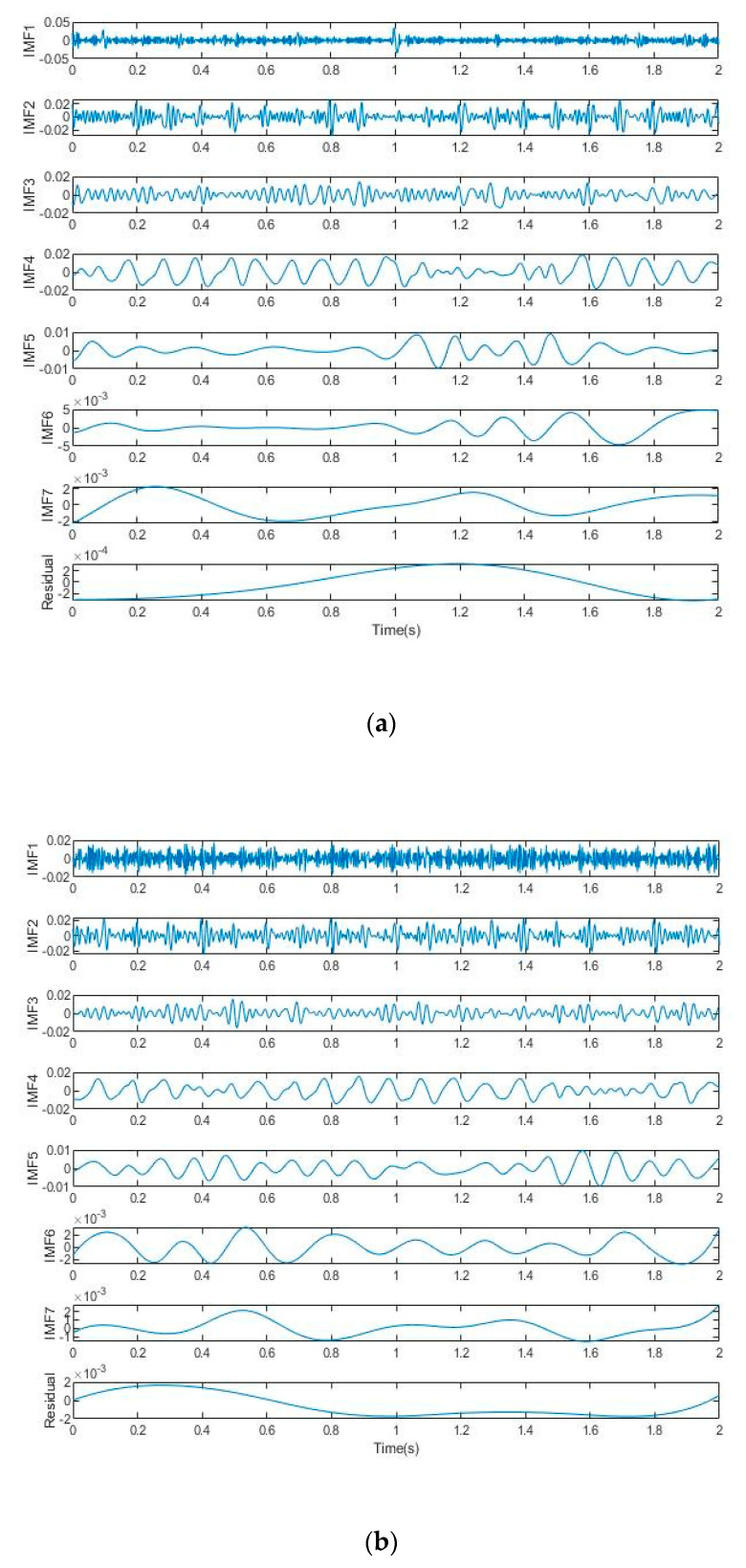

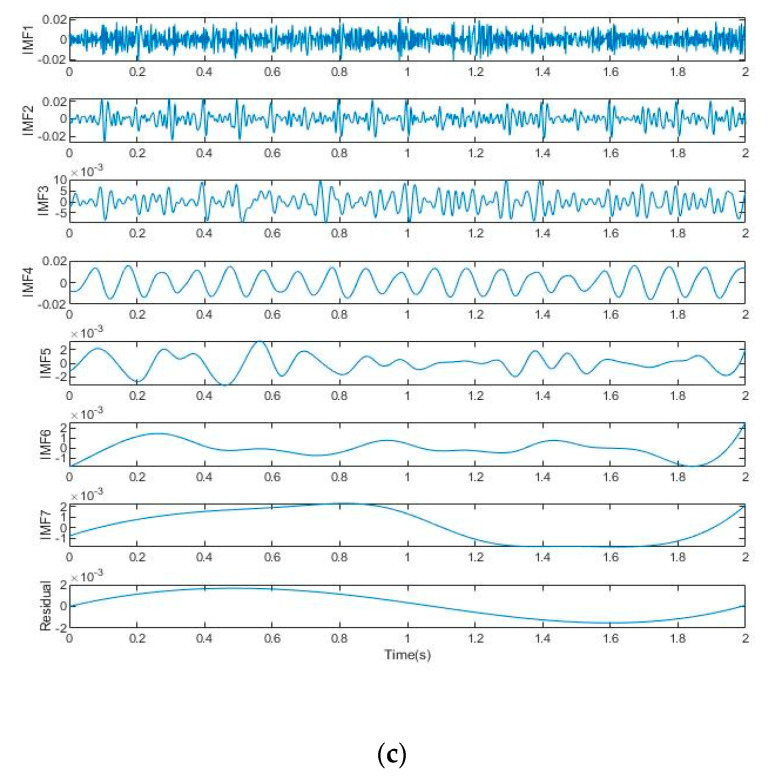
Decomposition diagram of noisy signal: (**a**) EMD decomposition; (**b**) EEMD decomposition; (**c**) CEEMD decomposition.

EMD, EEMD, and CEEMD decomposition are performed on the noisy signal, and the time domain diagram of each IMF is finally obtained, as shown in Figure 4. As can be seen from Figure 4, the IMF components obtained after EMD and EEMD decomposition always have the problem of modal aliasing, while the components obtained after CEEMD decomposition only contain one time scale characteristic component from IMF4, which effectively alleviates the problem of modal aliasing and is conducive to subsequent noise elimination.

**Figure 5:**
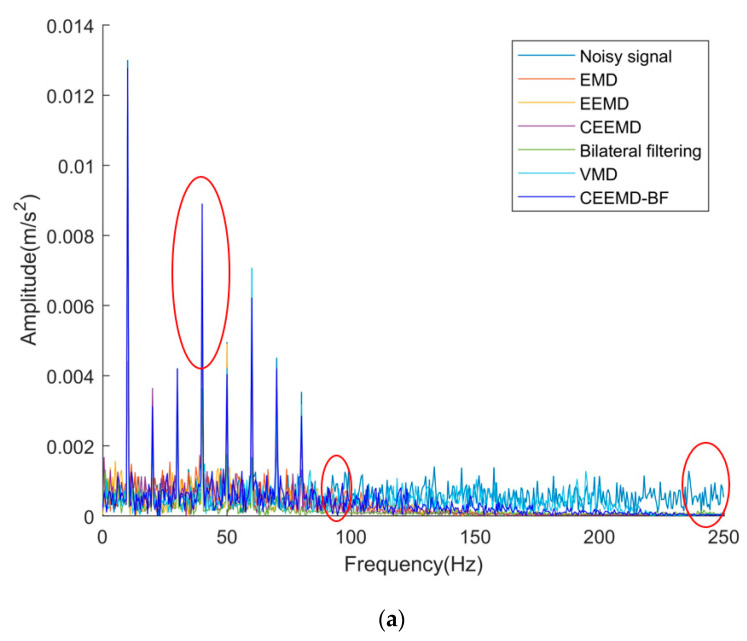

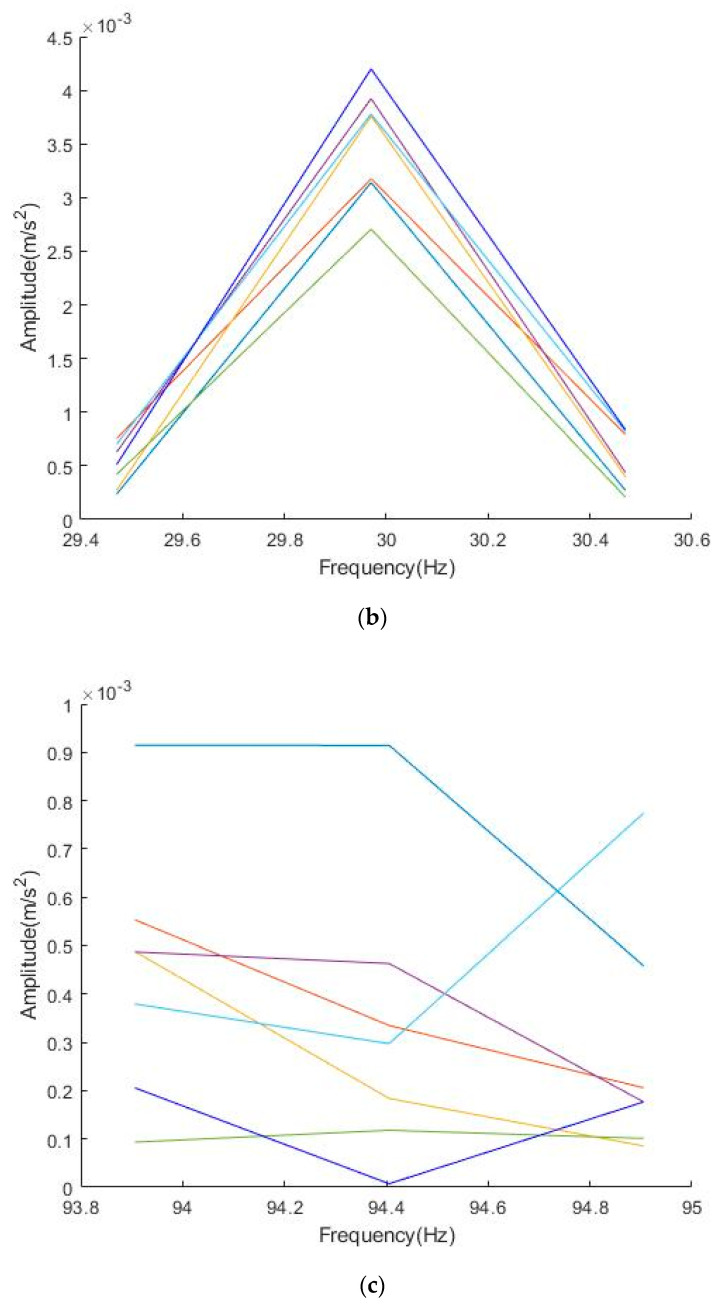

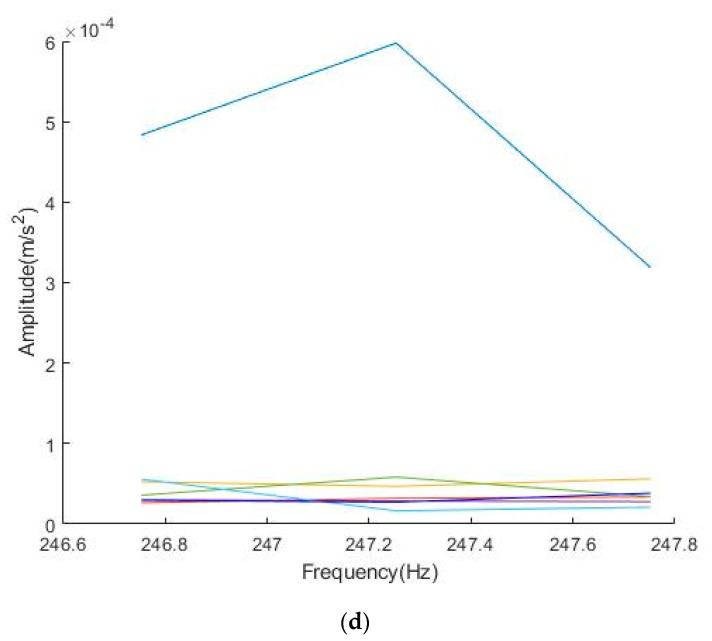
Frequency domain diagram after denoising: (**a**) global signals; (**b**) locally amplified signals-1; (**c**) locally amplified signals-2; (**d**) locally amplified signals-3.

We apply the newly proposed method (CEEMD-BF), EMD, EEMD, CEEMD, bilateral filtering, and VMD to the noisy elevator vibration signal. The overall denoising results are shown in Figure 5a. The amplifications of signals at the positions marked by the three red circles in Figure 5a are Figure 5b–d from left to right.

As can be seen from Figure 5, EMD, EEMD, CEEMD, bilateral filtering, VMD, and our newly proposed method can reduce the signal amplitude of medium and high frequency, that is, reduce or eliminate the medium- and high-frequency noise, but the top five methods will greatly reduce the signal amplitude at the main frequency, resulting in the loss of useful information in the signal. The method proposed in this paper can ensure that the amplitude of the main frequency signal is kept unchanged or slightly increased to the maximum extent, which is conducive to the further analysis and processing of the signal, and with the increase in noise frequency, the noise reduction effect of vibration signal obtained by using the method proposed in this paper is more obvious. Therefore, in general, the method proposed in this paper has a better denoising effect and is suitable for elevator signal denoising.

In order to express the denoising effect of different methods more intuitively in the form of data, we employ two evaluation indicators to judge the denoising effect. The evaluation indicators involved include signal-to-noise ratio and root mean square error. The SNR is equal to the effective power of the signal divided by the effective power of the noise, and the greater the value of SNR, the better the denoising effect. The RMSE is the square root of the variance between the original signal and the denoised signal, and the smaller the value of RMSE, the stronger the denoising ability.

(36) $$\mathrm{SNR}=10\log\left[\frac{\sum_{i=1}^{N}x_{i}^{2}}{\sum_{i=1}^{N}{\left(\hat{x_{i}}-x_{i}\right)}^{2}}\right]$$

(37) $$\mathrm{RMSE}=\sqrt{\frac{1}{N}\overset{N}{\underset{i=1}{\sum }}{\left(\hat{x_{i}}-x_{i}\right)}^{2}}$$

where *N* indicates the number of data, $x_{i}$ indicates pre-noised signals, and $\hat{x_{i}}$ indicates post-noised signals.

The final denoising evaluation index value is shown in Table 1. It can be seen from the table that compared with the other five methods, the signal-to-noise ratio of the denoised signal obtained by the method proposed in the paper is large, and the root mean square error is small, indicating that the denoising effect of this method is better.

In addition, in order to verify the denoising effect of CEEMD-BF under different SNR, we add different levels of noise to the signal to obtain the SNR and root mean square error of the denoised signal. The results are shown in Table 2.

As can be seen from Table 2, as the SNR increases, the SNR of the denoised signal becomes larger and larger, and the corresponding root mean square error becomes smaller and smaller. Therefore, the larger the SNR is, the better the denoising effect will be.

## 5. Elevator Vibration Signal Denoising

**Figure 6:**
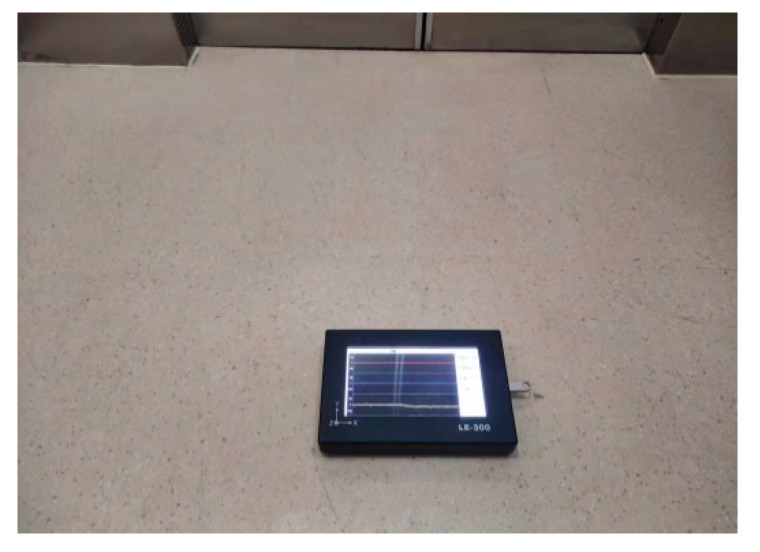
Signal acquisition device.

**Figure 7:**
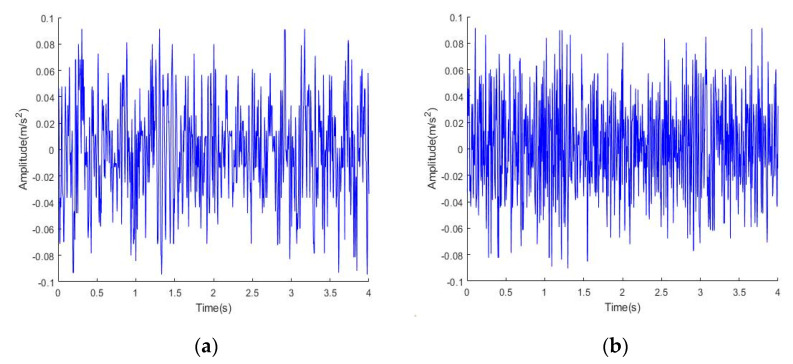
Elevator horizontal vibration signal: (**a**) *X*-axis vibration signal; (**b**) *Y*-axis vibration signal.

In order to verify the function of the method proposed in the paper in the denoising of elevator vibration signal, we collected the vibration signal of an elevator car. The equipment used to collect data in the experiment is LE-300, as shown in Figure 6. The signal acquisition frequency is 256 Hz, and the collected vibration signal in the horizontal direction of elevator is shown in Figure 7. Because the vibration signals of the *X*-axis and *Y*-axis of elevator vibration signal have little difference, the paper firstly focuses on the comparison and analysis of the vibration signals of the *X*-axis elevator and then verifies the denoising effect of the new method on the *Y*-axis signal. The number of points collected is 1024.

**Figure 8:**
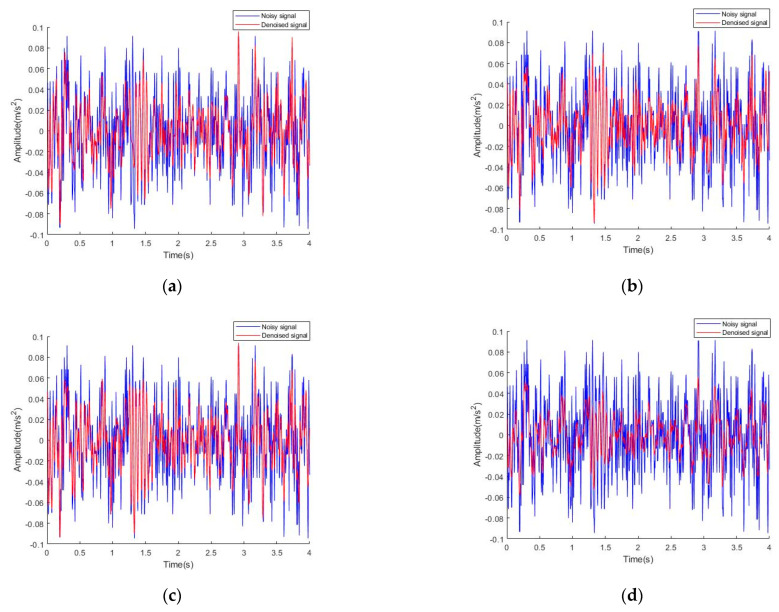

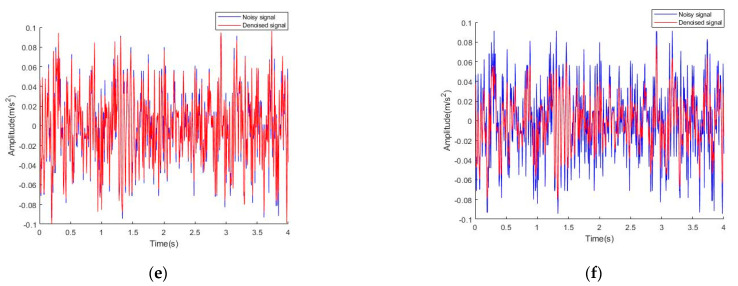
Time domain diagram of *x*-axis signal before and after denoising: (**a**) EMD denoising method; (**b**) EEMD denoising method; (**c**) CEEMD denoising method; (**d**) bilateral filtering denoising method; (**e**) VMD denoising method; (**f**) CEEMD-BF denoising method.

**Figure 9:**
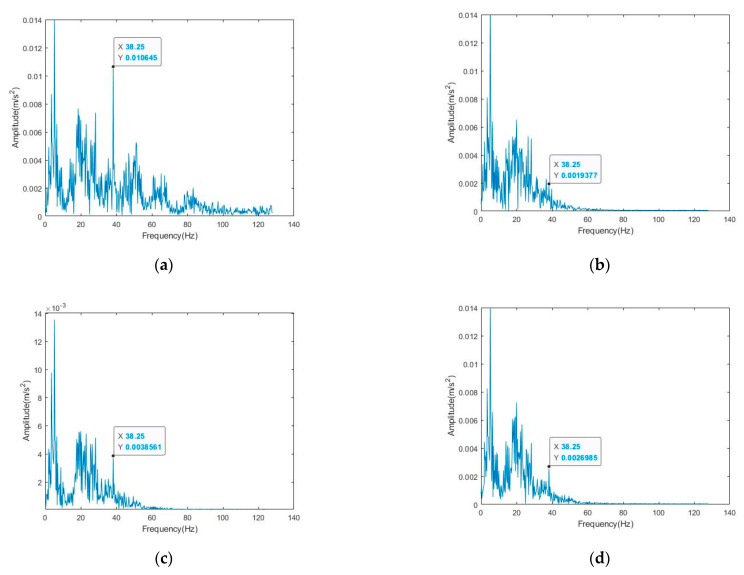

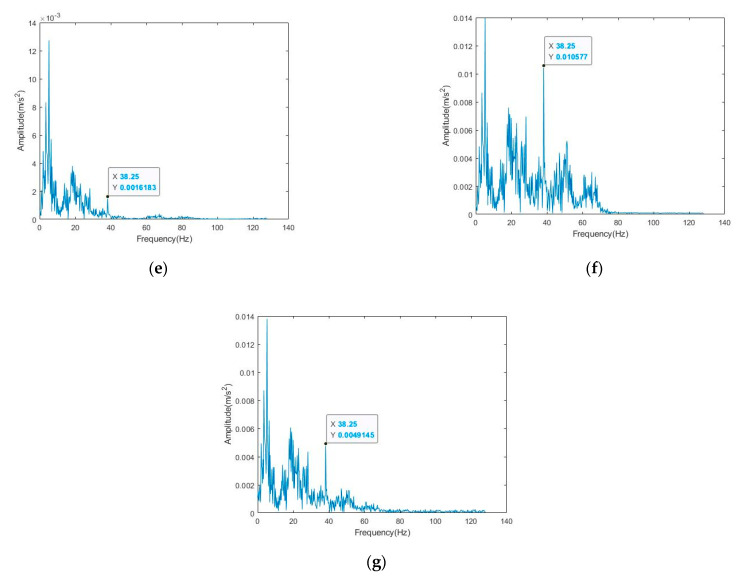
Frequency domain diagram of *x*-axis signal before and after denoising: (**a**) noisy signal; (**b**) EMD denoising method; (**c**) EEMD denoising method; (**d**) CEEMD denoising method; (**e**) bilateral filtering denoising method; (**f**) VMD denoising method; (**g**) CEEMD-BF denoising method.

We first use EMD, EEMD, CEEMD, bilateral filtering, VMD denoising, and the method proposed in the paper to denoise from the *X*-axis vibration signal. The time domain diagram and frequency domain diagram after denoising are shown in Figure 8 and Figure 9.

The horizontal vibration frequency of the elevator is generally 0.5~80 Hz. As can be seen from Figure 9a, the collected elevator vibration signals are mainly concentrated at 0.5~70 Hz. The vibration amplitude of the elevator car signal is larger at 5.25 Hz and 38.25 Hz, and other major frequency components are 18.5 Hz, 28.25 Hz, 51.25 Hz, and 65.25 Hz. As can be seen from Figure 9b–d, the denoising effects of vibration signals obtained by using EMD, EEMD, and CEEMD have no significant difference. When the frequency is close to 40 Hz, the amplitude of the vibration signal tends to 0, while the horizontal vibration frequency of the elevator vibration signal collected in the paper is 0.5~70 Hz. Therefore, these three denoising methods will lose some vibration information of the elevator. According to the time domain diagram in Figure 8d, it seems that the denoising effect of bilateral filtering is better than that of other methods. However, combined with the frequency domain diagram in Figure 9e, when the frequency is greater than 20 Hz, the amplitude of the main frequency decreases greatly. Therefore, in a comprehensive view, bilateral filtering filters out many useful signals and cannot ensure that the information of the original signal is not affected by the denoising operation. The VMD denoising in Figure 9f can remove the noise after 80 Hz, but has almost no effect on the noise before 80 Hz. There is no obvious difference between the time-domain graphs of the vibration signal before and after denoising in Figure 8e, so the denoising effect of the VMD de-noising method is not obvious and cannot be used for the denoising of elevator car vibration signals. Therefore, comprehensively, the method proposed in this paper can ensure that the amplitude at the main frequency of the signal is kept at a large value to the maximum extent, and the noise exceeding the normal vibration frequency range of the elevator can be effectively removed. The denoising results of the method proposed in this paper are good.

**Figure 10:**
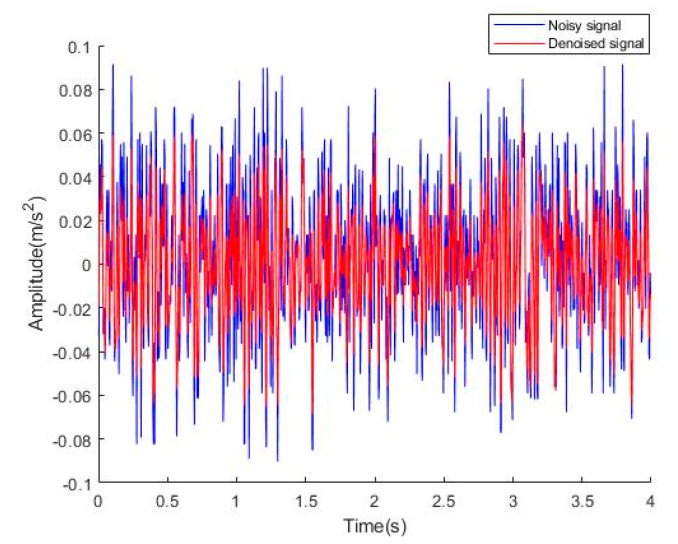
Time domain diagram of *y*-axis signal before and after denoising.

**Figure 11:**
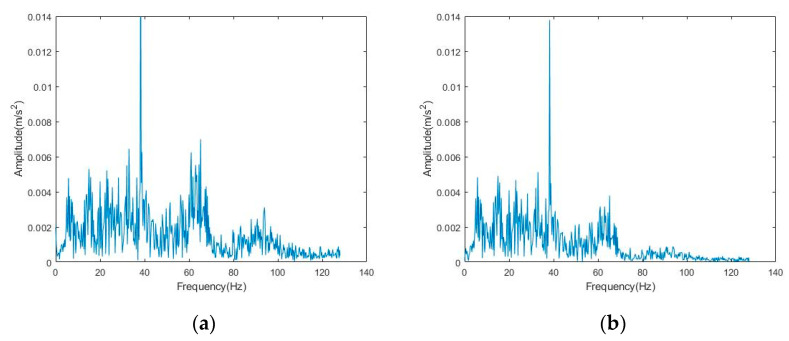
Frequency domain diagram of *y*-axis signal before and after denoising: (**a**) noisy signal; (**b**) CEEMD-BF denoising method.

We also applied the new method to the *y*-axis vibration signal of the elevator. The time-domain and frequency-domain diagrams before and after denoising are shown in Figure 10 and Figure 11. From the time-domain and frequency-domain diagrams, we can see that this method is not only applicable to the *x*-axis vibration signal denoising but also applicable to the *y*-axis vibration signal denoising. It can retain the useful information in the signal to the maximum extent, remove the noise, and obtain a good denoising effect.

## 6. Conclusions

In the paper, an elevator car vibration signal denoising method based on CEEMD and bilateral filtering is presented. Firstly, the method decomposes vibration signal into several inherent modal functions by CEEMD. CEEMD can better solve the problem of mode aliasing in signal decomposition. Next, the method determines the false components on the basis of computing the correlation coefficients. Then, the method determines the partial mean value of the multi-scale permutation entropy of the remaining components. If the partial mean value of IMF is large, it indicates that noise is the dominant component in the IMF and needs bilateral filtering to remove the noise. Finally, the vibration signal after denoising is obtained through signal reconstruction. The effectiveness and efficiency of this method are testified by simulation and experiment. Compared with EMD denoising, EEMD denoising, CEEMD denoising, bilateral filtering denoising, and VMD denoising, the method can not only retain the useful signal better but also filter out more noise. In the following research, we will focus on the signal processing speed of this method and how to achieve better results in the signal processing process of different specifications of elevators.

## Figures and Tables

**Table 1:** Evaluation index of denoising.

	Signal-to-Noise Ratio	Root Mean Square Error
CEEMD-BF	7.3515	0.0064
EMD denoising	5.1834	0.0074
EEMD denoising	5.1177	0.0074
CEEMD denoising	6.1136	0.0066
Bilateral filtering denoising	3.9266	0.0085
VMD denoising	4.9947	0.0075

**Table 2:** The denoising effect of CEEMD-BF under different noises.

Signal-to-Noise Ratio of Noise	Signal-to-Noise Ratio of Denoised Signal	Root Mean Square Error of Denoised Signal
60	13.1633	0.0033
55	13.0197	0.0033
50	12.4061	0.0036
45	10.9547	0.0042
40	7.3515	0.0064
35	2.8969	0.0107

## Data Availability

Not applicable.

## References

[1] Mallat S.G. (1989). A theory for multiresolution signal decomposition: The wavelet representation. IEEE Trans. Pattern Anal. Mach. Intell..

[2] Xu Y., Weaver J.B., Healy D.M., Lu J. (1994). Wavelet transform domain filters: A spatially selective noise filtration technique. IEEE Trans. Image Process. A Publ. IEEE Signal Process. Soc..

[3] Donoho D.L., Johnstone I.M. (1994). Ideal Spatial Adaptation by Wavelet Shrinkage. Biometrika.

[4] Li C.J., Yang Q.F., Zhou S.R., Li Z.J., Yang X.L. (2014). Signal Denoising Based on Slip Threshold Value of Wavelet Packets. Appl. Mech. Mater..

[5] Chen X., Lin G., Zhang Y. (2014). Denoising Method Based on Sparse Representation for WFT Signal. J. Sens..

[6] Huang N.E., Shen Z., Long S.R., Wu M.C., Shih H.H., Zheng Q., Yen N.-C., Tung C.C., Liu H.H. (1998). The empirical mode decomposition and the Hilbert spectrum for nonlinear and non-stationary time series analysis. Proc. Math. Phys. Eng. Sci..

[7] Falco C.M., Chang C.-C., Jiang X., Wu W., Peng H. A new denoising approach based on EMD. Proceedings of the Sixth International Conference on Digital Image Processing (ICDIP 2014).

[8] Wu Z., Huang N.E. (2009). Ensemble empirical mode decomposition: A noise-assisted data analysis method. Adv. Adapt. Data Anal..

[9] Yeh J.-R., Shieh J.-S., Huang N.E. (2011). Complementary Ensemble Empirical Mode Decomposition: A Novel Noise Enhanced Data Analysis Method. Adv. Adapt. Data Anal..

[10] Dang S., Tian W., Qian F. (2011). EMD- and LWT-based stochastic noise eliminating method for fiber optic gyro. Measurement.

[11] Chegini S.N., Bagheri A., Najafi F. (2019). Application of a new EWT-based denoising technique in bearing fault diagnosis. Measurement.

[12] Zuo L.-Q., Sun H.-M., Mao Q.-C., Liu X.-Y., Jia R.-S. (2019). Noise Suppression Method of Microseismic Signal Based on Complementary Ensemble Empirical Mode Decomposition and Wavelet Packet Threshold. IEEE Access.

[13] Yan Y., Xing H. (2021). Small Floating Target Detection Method Based on Chaotic Long Short-Term Memory Network. J. Mar. Sci. Eng..

[14] Zhang X., Zheng Z., Wu X. (2020). A novel regional annual precipitation predicting model. Desalination Water Treat..

[15] Xing H., Yan Y. (2018). Detection of Low-Flying Target under the Sea Clutter Background Based on Volterra Filter. Complexity.

[16] Li J., Cai J., Tang J.-T., Li G., Zhang X., Xu Z.-M. (2019). Magnetotelluric signal-noise separation method based on SVM–CEEMDWT. Appl. Geophys..

[17] Wang J., He X., Ferreira V.G. (2015). Ocean Wave Separation Using CEEMD-Wavelet in GPS Wave Measurement. Sensors.

[18] Dragomiretskiy K., Zosso D. (2014). Variational Mode Decomposition. IEEE Trans. Signal Process..

[19] Long J.C., Wang X.P., Dai D.D., Tian M., Zhu G.W., Zhang J. (2017). Denoising of UHF PD signals based on optimised VMD and wavelet transform. IET Sci. Meas. Technol..

[20] Yu S.W., Ma J.W. (2018). Complex Variational Mode Decomposition for Slop-Preserving Denoising. IEEE Trans. Geosci. Remote Sens..

[21] Zhang M., Gunturk B.K. (2008). Multiresolution bilateral filtering for image denoising. IEEE Trans Image Process..

[22] Yu H., Zhao L., Wang H. (2009). Image denoising using trivariate shrinkage filter in the wavelet domain and joint bilateral filter in the spatial domain. IEEE Trans Image Process.

[23] Wu G., Luo S., Yang Z. (2020). Optimal weighted bilateral filter with dual-range kernel for Gaussian noise removal. IET Image Process..

[24] Lei Y., Lin J., He Z., Zuo M.J. (2013). A review on empirical mode decomposition in fault diagnosis of rotating machinery. Mech. Syst. Signal Process..

[25] Gu J., Peng Y.X. (2021). An improved complementary ensemble empirical mode decomposition method and its application in rolling bearing fault diagnosis. Digit. Signal Prog..

[26] Civera M., Surace C. (2021). A Comparative Analysis of Signal Decomposition Techniques for Structural Health Monitoring on an Experimental Benchmark. Sensors.

[27] Nassef M.G.A., Hussein T.M., Mokhiamar O. (2021). An adaptive variational mode decomposition based on sailfish optimization algorithm and Gini index for fault identification in rolling bearings. Measurement.

[28] Shreyamsha Kumar B.K. (2013). Image denoising based on gaussian/bilateral filter and its method noise thresholding. Signal Image Video Process..

[29] Tomasi C., Manduchi R. (1998). Bilateral filtering for gray and color images. Proceedings of the 6th International Conference on Computer Vision.

[30] Liu N., Schumacher T. (2020). Improved Denoising of Structural Vibration Data Employing Bilateral Filtering. Sensors.

